# Effect of Bovine Serum Albumin (BSA) Concentration on Cryopreservation of Booroolong Frog Sperm with Evaluation of Post-Thaw Motility in Caffeine

**DOI:** 10.3390/vetsci12010030

**Published:** 2025-01-08

**Authors:** Zara M. Anastas, Aimee J. Silla, Phillip G. Byrne, Rebecca J. Hobbs, Michael S. McFadden, Jonathan Daly, Justine K. O’Brien

**Affiliations:** 1 Environmental Futures, School of Earth, Atmospheric and Life Sciences, University of Wollongong, Wollongong, NSW 2522, Australia; zanastas@uow.edu.au (Z.M.A.); asilla@uow.edu.au (A.J.S.); pbyrne@uow.edu.au (P.G.B.); 2 Taronga Institute of Science and Learning, Taronga Conservation Society Australia, Mosman, NSW 2088, Australia; rhobbs@zoo.nsw.gov.au (R.J.H.); mmcfadden@zoo.nsw.gov.au (M.S.M.); jondaly@zoo.nsw.gov.au (J.D.); 3 School of Biological, Earth and Environmental Sciences, University of New South Wales, Sydney, NSW 2033, Australia

**Keywords:** amphibian, sperm cryopreservation, bovine serum albumin, caffeine, conservation, breeding program, reproductive technologies, spermic urine, biobanking, biorepository

## Abstract

Reproductive technologies, including sperm cryopreservation, offer conservationists enhanced capacity to genetically manage populations and improve the outcomes of conservation breeding programs (CBPs). Despite this potential, the post-thaw quality of amphibian sperm is highly variable following cryopreservation, and research focused on protocol refinement is needed. The aim of this study was twofold: (1) to investigate the effect of the addition of bovine serum albumin (BSA) to the cryopreservation medium (pre-freeze), and (2) the effect of the addition of caffeine to the activation medium (post-thaw), on post-thaw sperm characteristics in the critically endangered Booroolong frog (*Litoria booroolongensis*). Spermic urine samples were collected from 14 male frogs following hormonal induction of spermiation, and each sample was split among three cryopreservation treatments, where the cryopreservation medium contained either 0 (control), 0.5, or 1% BSA (*w*/*v*). Samples were cryopreserved and thawed, and sperm motility was then activated in one of two activation treatments: Milli-Q water (control) or Milli-Q water plus 4.5 mM caffeine. Sperm viability (proportion live/dead) was assessed using fluorescent microscopy, and sperm motility metrics were evaluated using computer-assisted sperm analysis (CASA). Results from this study showed that BSA concentration had no effect on post-thaw sperm viability. Additionally, neither BSA concentration nor activation in caffeine influenced post-thaw sperm motility characteristics (total motility, forward progressive motility, and velocity). Assessment time of sperm motility varied from 5 to 13 min post-activation and was significantly correlated with each motility measure, with motility and velocity metrics decreasing as time post-activation increased. The results reported herein provide no evidence for an effect of BSA or caffeine at the concentrations tested on post-thaw sperm characteristics in the Booroolong frog, but they highlight the time-sensitive nature of sperm assessment post-thaw and implications for the timing of sperm handling during assisted fertilisation efforts.

## Simple Summary

The application of reproductive technologies has the potential to improve conservation breeding program (CBP) outcomes. Biorepositories can aid in securing and maintaining the genetic diversity of captive populations and the wild populations they aim to recover. However, research is needed to optimise the post-thaw quality of stored amphibian sperm and in turn maximise the efficient use of cryopreserved samples. Here, we investigated the effect of bovine serum albumin (BSA) and caffeine on sperm characteristics after freezing and thawing in the critically endangered Booroolong frog, *Litoria booroolongensis*. Using spermic urine samples from 14 males, we found that neither BSA nor caffeine influenced post-thaw sperm quality at the concentrations tested.

## 1. Introduction

In response to the amphibian extinction crisis, conservation breeding programs (CBPs), which aim to establish genetically representative, self-sustaining colonies to provide a means of bolstering wild populations, have been established for a range of threatened species [1,2,3,4,5,6]. Program success is dependent on a number of factors, including the provision of captive environmental conditions suitable for breeding and offspring rearing, generation of viable individuals for wild release, maintenance of genetic diversity, and continued funding and resources [7]. Reproductive technologies, which encompass a range of tools that have the potential to both bolster propagation and improve genetic management of species within CBPs, are increasingly being utilised to assist these programs to reach propagation and genetic targets and enhance species recovery [8,9,10,11]. Sperm cryopreservation has long been heralded as a tool for improving CBPs by allowing the storage of living genetic material in biorepositories [12,13]. These biorepositories offer conservationists the ability to genetically manage populations by facilitating the exchange of genetic material among both ex situ and in situ populations, in addition to increasing the availability of genetic material well beyond the lifespan of individual animals [8,9,10,14].

Though there are clear benefits of sperm cryopreservation and its integration into threatened species management, further research is required to optimise protocols to promote post-thaw sperm quality [15]. The process of cryopreserving sperm cells is impactive, and not all cells survive the freeze–thaw process. For amphibians, sperm death or damage is highly variable among species, and cryopreservation has resulted in a loss of between 10 to 90% of pre-freeze motility after thawing [16]. This loss is derived from functional changes to sperm cell membranes and organelles [17,18,19,20] and highlights the need for species-specific protocol optimisation to minimise cryoinjury. Whilst previous studies in amphibians have optimised protocols through modification of cryopreservation media and cooling/thawing rates, post-thaw sperm outcomes may be further improved through supplementation with protective and stimulatory additives.

A range of additives to cryopreservation and thawing media have been trialled in various taxa, often selected based on their ability to act as antioxidants and protect sperm cells from cryoinjury. Antioxidants may improve cryopreservation outcomes by minimising the production of reactive oxygen species (ROS). Whilst sperm cells naturally produce ROS as a by-product of cellular metabolism, high levels of ROS can cause oxidative stress that canreduce cell functioning and cause cellular damage, or cell death [21,22,23,24]. The supplementation of the cryopreservation medium with antioxidants has proven to be beneficial to the post-thaw sperm quality of various mammalian [24,25,26] and fish species [27,28,29,30,31]. One antioxidant that is commonly employed in sperm cryopreservation studies is bovine serum albumin (BSA), a protein derived from cows [32]. In addition to its ability to scavenge ROS and thus minimise oxidative stress [33,34], BSA has several properties that are linked to improved post-thaw sperm viability and motility. Specifically, it has been reported that BSA can have beneficial effects by increasing membrane fluidity [35], maintaining acrosome integrity [33], reducing DNA damage [36], and increasing intracellular ATP content [37]. Supplementation with BSA has resulted in improved post-thaw sperm metrics in several taxa, including mammals [34,38], reptiles [39], and fish [40,41]. In amphibians, while several studies have included BSA in cryopreservation media, few have empirically tested the effect of varying concentrations of BSA on post-thaw sperm characteristics [42,43,44,45,46], and findings have been variable. In the Eastern tiger salamander (*Ambystoma tigrinum*), the addition of 0.5% BSA to a 5% DMSO cryopreservation medium did not significantly affect sperm motility compared to the addition of 5% trehalose or 5% sucrose [45]. Additionally, in a study in the yellow-spotted mountain newt (*Neurergus derjugini*), whilst no post-thaw motility was observed when sperm were cryopreserved in either 10% DMSO with 1% BSA or 5% DMSO with 5% BSA, there was 31% post-thaw motility following cryopreservation in 10% DMSO with 5% BSA [46]. In a recent study in an anuran, the Fowler’s toad (*Anaxyrus fowleri*), Burger and colleagues [42] theorised that the improved post-thaw sperm motility reported, compared to a previous study in the same species [47], may have been attributed to the addition of 0.5% BSA to the cryopreservation medium. For optimisation of protocols for the cryopreservation of amphibian sperm, empirical studies directly examining the influence of BSA on sperm function post-thaw in a greater diversity of amphibian species are needed.

Whilst including additives in cryopreservation media pre-freeze may minimise cryo-injury to result in a greater number of viable sperm post-thaw, optimisation of the activation medium may facilitate enhanced motility activation of the viable sperm that have survived the freeze–thaw process. In species where fertilisation occurs externally within freshwater (freshwater teleosts and amphibians), sperm motility is typically activated through a sudden decrease in osmolality [48,49]. To decrease the osmolality of the sperm suspensions, which would otherwise be high due to the cryoprotectants, cryopreserved sperm is immediately diluted after thawing [50]. The mechanism behind sperm motility activation in amphibians is the increase in intracellular cyclic adenosine monophosphate (cAMP) that is triggered by the decrease in osmolality [51]. Amphibian sperm motility activation is theorised to be able to be further enhanced by inhibiting phosphodiesterase (PDE) activity, as PDEs are enzymes which are involved in the regulation of intracellular cAMP [52] which can be achieved through the addition of PDE inhibitors such as caffeine, pentoxifylline, and theophylline. In addition to having a stimulatory effect, caffeine may act as an antioxidant [53], scavenging ROS that may have accumulated before and following the freezing and thawing process. Thus, the addition of caffeine to the post-thaw activation medium may enhance post-thaw sperm motility via multiple pathways.

Caffeine has been added to the post-thaw activation media and resulted in increased post-thaw sperm performance in a range of taxa, including several mammals [54,55] including humans [56,57,58], reptiles [39,59], corals [60,61], and fish [62,63]. In amphibians, no study to date has tested the effect of adding PDE inhibitors such as caffeine to the activation medium of frozen–thawed sperm; however, three studies have tested their effect in fresh sperm samples. In a study in the cane toad *Rhinella marina*, there was a significant increase in sperm motility with increasing theophylline concentration [64]. Conversely, in a study with the Puerto Rican frog, *Eleutherodactylus coqui*, caffeine at a final concentration of 3 mM added to fresh sperm suspensions was associated with a non-significant decrease in total sperm motility [65]. In a study of the Australian Booroolong frog, *Litoria booroolongensis*, the addition of 2.5 mM or 5 mM caffeine did not influence initial percent sperm motility or velocity, however, samples containing caffeine at 2.5 mM displayed an increase in both performance measures several hours post-activation [66]. The results of the study on fresh Booroolong frog sperm, in addition to the numerous positive results reported on frozen–thawed sperm of other taxa, highlight the potential for caffeine to improve the post-thaw motility of amphibian sperm, justifying empirical investigation.

The aim of this study was twofold: (1) to investigate the effect of the addition of two concentrations of BSA to the cryopreservation medium (pre-freeze), and (2) the effect of caffeine addition to the activation medium (post-thaw), on post-thaw sperm performance (sperm viability and motility) in the Booroolong frog, *Litoria booroolongensis*.

## 2. Materials and Methods

### 2.1. Study Species

The Booroolong frog (*L. booroolongensis*) is a medium-sized (snout-to-vent length; male = 30 to 42 mm and female = 40 to 58 mm) riverine species, restricted to remnant populations inhabiting permanent rocky streams along the Great Dividing Range in New South Wales and north-eastern Victoria [4,67,68]. This species has a relatively short lifespan of approximately four years, which exacerbates the effects of stochastic water availability on species’ persistence [4,69]. This species is currently listed as endangered under both state and federal legislation in Australia, as well as by the International Union for the Conservation of Nature [70]. In response to drought conditions threatening remaining populations in NSW, a captive breeding and release program was established at Taronga Zoo (Sydney, NSW, Australia) with the collection of founder animals from the northern extent of their range in late 2019 in consultation with the Department of Climate Change, Energy, the Environment and Water, NSW, Australia [50].

### 2.2. Animals

Booroolong frogs used in the present study were two-year-old first-generation (F1) adult males (mass ranged from 3.92 to 6.11 g; mean mass (±SEM) = 4.85 ± 0.16 g, *n* = 14), bred and reared at Taronga Zoo from field-caught parents (collected as part of an emergency intervention from the Northern Tablelands of New South Wales, Australia). Frogs were housed in same-sex groups (four to six individuals) in glass terrariums fitted with mesh canopies (48 long × 60 wide × 42 high cm, inclusive of canopy) with sediment and carbon-filtered water, artificial plants, and two to three rocks for refuge. Males were fed crickets dusted with calcium and multivitamin powder (Repashy Calcium Plus; Repashy, Oceanside, CA, USA) two times per week. At the time of the study, lighting was provided from approximately 7 a.m. to 7 p.m. via 5.0 UVB fluorescent tubes (ReptiSun^®^ T5; ZooMed Laboratories, San Luis Obispo, CA, USA) placed directly above the terrariums, which were connected to a photoelectric cell to provide a lighting cycle that reflected the natural photoperiod outside of the facility. The temperature within the room was programmed to cycle between 24 and 27 °C during the day and 18 and 20 °C at night.

### 2.3. Split-Sample Experimental Design

**Figure 1:**
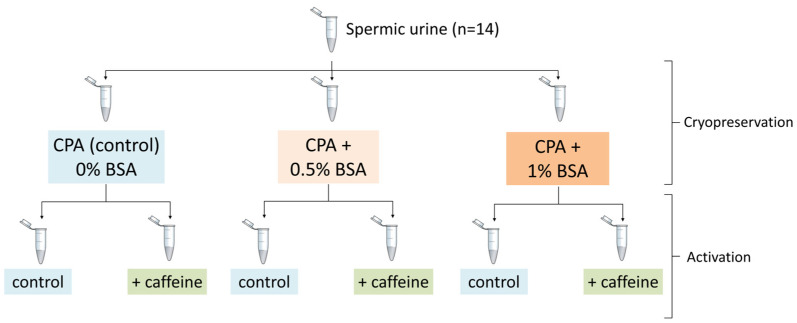
Split-sample experimental design. Shown is each spermic urine sample (*n* = 14), collected from male Booroolong frogs (*Litoria booroolongensis*), evenly divided among three cryopreservation treatments: cryoprotective agents (CPA) with 0% bovine serum albumin (BSA), CPA with 0.5% BSA, and CPA with 1% BSA. After thawing, these samples were then split among two activation treatments: Milli-Q water (control activation) and Milli-Q water with 4.5 mM caffeine (caffeine activation).

To test the effect of BSA addition (0%, 0.5%, or 1% *w*/*v* BSA) to the cryoprotectant medium (CPA), as well as the effect of the addition of caffeine to the activation medium, on post-thaw sperm characteristics, a split-sample experimental design was adopted. This approach involved evenly dividing the sperm suspensions from each individual male across all experimental treatments (Figure 1). Split-sample designs are routinely adopted by studies quantifying sperm performance responses in order to control for individual male variation in sperm quality. First, spermic urine samples were collected according to the methods described below, one sperm sample per individual male (*n* = 14). Each sperm sample was evenly divided among the three BSA treatments before cryopreservation. Cryopreserved straws were held in liquid nitrogen storage (−196 °C) for 11 to 14 weeks before thawing. Upon thawing, each cryopreserved straw sample was evenly divided into two subsamples, one diluted in a control motility activation medium (Milli-Q water, 0 mOsmol kg^−1^) and the other in an activation medium supplemented with caffeine (4.5 mM, 2.5 mOsmol kg^−1^). The osmolalities of activation solutions were determined using a freezing point depression osmometer (Osmo1^®^ Advanced Instruments LLC, Norwood, MA, USA). The experimental design employed resulted in six distinct experimental treatments: (1) CPA (control) 0% BSA, control activation; (2) CPA (control) 0% BSA, caffeine activation; (3) CPA + 0.5% BSA, control activation; (4) CPA + 0.5% BSA, caffeine activation; (5) CPA + 1% BSA, control activation; (6) CPA + 1% BSA, caffeine activation (Figure 1). Experimental procedures were conducted from 21 to 23 November and 12 to 13 December 2023 for spermic urine collection and cryopreservation, and from 28 February to 1 March 2024 for thawing and activation.

### 2.4. Spermic Urine Collection and Pre-Freeze Assessment

Spermic urine collections occurred over five days, and on each day, spermic urine was collected from four to eight males (14 males total). Methods for hormonal induction and spermic urine collection followed protocols that have previously been refined for this species [71]. Males received a standard hormone dose of 80 IU purified human chorionic gonadotropin (hCG; Chorulon^®^, MSD Animal Health, UK Ltd., Milton Keynes, UK) diluted in 100 µL simplified amphibian ringer (3.6 mM of sodium bicarbonate, 112.5 mM of sodium chloride, 2.0 mM of potassium chloride, and 1.35 mM of calcium chloride; pH 7.39; 223 mOsmolkg^−1^, measured using a freezing point depression osmometer [Osmo1^®^ Advanced Instruments LLC, Norwood, MA, USA]), injected subcutaneously into the dorsal lymph sac using ultra-fine, sterile 31-gauge needles. Following hormone injection, males were weighed to the nearest 0.01 g using a digital scale and then placed into individual ventilated specimen containers (6 cm high × 4 cm diameter; holes were made into the lids for ventilation), each with one Kimtech^®^ Kimwipe tissue added, wetted with 10 mL of reverse osmosis (RO) water to provide adequate hydration to allow hourly spermic urine collection. Spermic urine was collected by inserting a 50 µL microcapillary tube (Micro-caps, Drummond Scientific Co., Broomall, PA, USA; fire-polished and cooled) into the cloaca to stimulate urination. Spermic urine collections occurred every hour for eight to 11 h post-injection, and samples were pooled for each individual. Pooled samples were kept at 4 °C in an insulated semen shipping container (Equitainer II, Hamilton Research, South Hamilton, MA, USA) for the duration of collections until pre-freeze assessment and cryopreservation. After the final collection time point, the volume of each pooled sample was measured. Spermic urine volume ranged from 72 to 239 µL (mean volume ± SEM = 124 ± 12.6 µL; *n* = 14).

Each sperm sample was homogenised, and a total of 7 µL was removed for the assessment of pre-freeze sperm quality (sperm concentration, viability, percent motility, and velocity). Sperm characteristics were assessed within a temperature-controlled laboratory set to 22 °C. Sperm viability (proportion live/dead) was assessed using a live/dead sperm viability kit (LIVE/DEAD™ Sperm Viability Kit, Molecular Probes Inc., Eugene, OR, USA). A 2 µL aliquot of the homogenised sperm sample was incubated in the dark in 17 µL SYBR-14 (diluted 1:1000 in Milli-Q water) for five minutes, after which 1 µL propidium iodide (PI, 2.4 mM) was added, and the sample incubated in the dark for an additional one minute (total volume 20 µL; 2 µL sperm sample + 18 µL stains). The sample was then loaded onto a Neubauer haemocytometer (0.1 mm depth; Brightline; Hawksley & Sons Ltd., Sussex, UK) and mounted on a fluorescent microscope (Motic BA310 Epi-LED FL microscope with a 470 nm long-pass filter; Motic Inc. Ltd., Causeway Bay, Hong Kong). Sperm viability was immediately assessed by counting sperm cells fluorescing green as viable (membrane-intact) and sperm cells fluorescing red as non-viable (membrane-damaged). To ensure accurate viability data, counts were performed for a total of 100 sperm cells, and from this, the percent viable cells was determined for each sample. Immediately following this, sperm concentration was determined from the same slide mount under brightfield at ×400 magnification with phase contrast by counting the number of cells present in 25 quadrats, which was then multiplied by the dilution factor (10). Sperm concentration in each sample ranged from 1.8 to 28.8 × 10^6^ sperm cells mL^−1^.

Sperm motility was assessed using a computer-assisted sperm analysis (CASA) system. A 3 µL aliquot of each sperm sample was loaded into a disposable semen analysis slide (SC20-01-04-B, 20 µm depth, Leja, Niew-Vennep, The Netherlands) and mounted onto a Zeiss microscope (Axiolab 5, Carl Zeiss Microscopy GmbH, Oberkochen, Germany) equipped with a 10 × negative phase contrast objective (Zeiss 10× NH CEROS II 160 nm), connected to a CASA CEROS II system (Animal Breeder, software version: v1.11.5; Hamilton Thorne Inc., Beverly, MA, USA). A minimum of 90 sperm cells were counted, with a mean of 105 sperm cells counted. The CASA system assessed total motility (%), forward progressive motility (FPM; %), average path velocity (VAP; µms^−1^), and curvilinear velocity (VCL; µms^−1^). Sperm motility assessment was conducted using CASA settings previously refined for this species and detailed elsewhere (see Hobbs, Upton, Calatayud, Silla, Daly, McFadden, and O’Brien [50]). A total of 12 out of 14 male replicates were assessed for sperm motility pre-freeze, as one sample was missed and one sample was loaded onto the incorrect slide type, and the corresponding male replicates were therefore excluded from pre-freeze characteristic means. Pre-freeze sperm characteristics are presented in Table 1.

### 2.5. Cryopreservation

Pooled spermic urine samples were split among the three cryopreservation medium treatments. To ensure all sample treatments were incubated with cryoprotectant for the same amount of time, samples from only one to two male replicates were handled for cryopreservation at one time—hereafter termed a ‘cryopreservation run’. Males were randomly allocated to a cryopreservation run, and within each cryopreservation run, the order of addition of cryoprotectant was randomised among males and treatments. Additionally, the order of cryopreservation medium treatments within male replicates was randomised in the same way. Spermic urine samples were homogenised before the removal of three aliquots of 20 or 25 µL per sample. Each of the three sperm subsamples was diluted 1:1 in pre-cooled cryopreservation medium (4 °C), corresponding with each of the three cryopreservation medium treatments (0%, 0.5%, or 1% BSA). The constituents of each of the three cryopreservation media are detailed in Table 2. The osmolalities of the cryopreservation media were determined using a vapour pressure osmometer (Vapro^®^ model 5600 Wescor Inc., South Logan, UT, USA), owing to the interference by the cryoprotectants with the freezing point depression osmometer. The pH values of the cryopreservation media were measured using a pH meter (Orion Star A211 pH meter, Thermo Fisher Scientific, Waltham, MA, USA). Cryopreservation media were added to the sperm subsamples in a stepwise fashion, with the sample homogenised via gentle agitation after the addition of each drop of diluent, such that the dilution was complete after 10 min. The diluted samples were then incubated for a further 10 min at 4 °C whilst they were loaded into 0.25-mL cryostraws (IMV technologies, l’Aigle, France). To do this, cryostraws were first loaded with a predetermined volume of SAR to counterweight the straw, then samples were homogenised by gentle agitation and loaded into the end of the cryostraws before being sealed with a metal ball bearing, ensuring an air gap was present between the SAR and the sample. A total of 42 cryostraws were prepared, *n* = 14 straws per treatment. Immediately following the 10 min incubation time, cryostraws were frozen using a dry shipper protocol described previously [50], which was initially adapted from protocols developed by Langhorne [72] and Roth et al. [73]. The protocol used in the present study involved placing the cryostraws into a pre-cooled 13 mm diameter goblet attached to a labelled aluminium storage cane, which was placed into a pre-cooled dry-shipper canister insert. The canister was then lowered into a charged dry shipper (Model CXR100, Worthington Enterprises, Columbus, OH, USA) such that the top of the canister was flush with the top of the neck of the dry shipper for 30 s, after which time the canister was then gently lowered into the bottom of the dry shipper, which was then covered and left to freeze for a minimum of 10 min before the samples were transferred to a liquid nitrogen dewar for long-term storage. The cooling rates were monitored with a thermocouple probe placed inside a 0.25 mL cryostraw containing CPA + 0% BSA; the average cooling rate over seven freezing runs, measured from +4 to −90 °C, was −14.6 °C ± 0.9 °C min^−1^. Excess spermic urine was cryopreserved for accession into the existing biorepository after completion of experimental cryopreservation runs using the control cryopreservation medium (0% BSA) and the same cooling protocol. Straws containing the cryopreserved samples were stored in liquid nitrogen for 11 to 14 weeks before thawing and post-thaw sperm quality assessment.

### 2.6. Post-Thaw Sperm Activation and Assessment

Cryostraws were thawed, and samples were activated and assessed over three consecutive days, from the 28 February to 1 March 2024. The order of thawing for male replicates was randomised using a random number generator before thawing, and the three cryostraws per male replicate (one for each of the three cryopreservation treatments) were thawed in succession, with the order of thawing additionally randomised in the same way. Cryostraws were thawed by removing the straw from the liquid nitrogen and holding it in air (air temperature ranged 21–22.9 °C; mean ± SEM 21.99 ± 0.05 °C; room temperature set to 22 °C) for two seconds, before submerging the straw for 5 s in a water bath (DairyMac rechargeable semen thaw flask, Genetics Australia, Bacchus Marsh, Australia) prefilled with SAR and set to 40 °C (temperature range = 39.1–40.2 °C). Post-thaw, straws were wiped with a Kimwipe to remove any condensation and SAR, and using a pair of sterilised scissors and whilst holding the straw horizontally, straws were then cut at the air gap and at the metal ball bearing, and a pipette was used to expel the sample into a 1.5 mL microcentrifuge tube (Eppendorf safelock, Thermo Fisher Scientific, Melbourne, Australia).

The thawed sperm sample was immediately homogenised by gently flicking the microcentrifuge tube and 5 µL removed for the assessment of sperm viability (proportion live/dead). Sperm viability was assessed by adding fluorescent nucleic acid stains; first, 4 µL SYBR-14 (diluted 1:50, SYBR-14:SAR, *v*/*v*) was added to the 5 µL aliquot of sperm sample, and the solution was incubated in the dark for five minutes, after which 1 µL propidium iodide (PI; 2.4 mM) was added, and the sample incubated in the dark for an additional one minute (total volume 10 µL; 5 µL sperm sample +5 µL stains). The dilution ratio of sperm-to-dyes was reduced post-thaw compared to pre-freeze sperm viability methods (see Section 2.4) to limit further dilution of sperm concentration (as samples had already been diluted with the addition of CPA). This ensured that the proportion of live/dead sperm could be assessed for a total of 100 sperm cells.

To activate sperm motility, the sample was divided among two 1.5 mL microcentrifuge tubes (10 µL per tube) and immediately activated in the two treatments: control (Milli-Q water only) or caffeine (final concentration 4.5 mM caffeine in Milli-Q water). The sample was diluted 1:4 (10 µL sperm sample plus 40 µL activation medium) to lower the osmolality and stimulate sperm motility. The activation medium was added in a stepwise fashion (four aliquots of 10 µL) with gentle flicking of the microcentrifuge tube between aliquot additions. Samples were diluted in activation medium after thawing to minimise the potential cytotoxic effects of the cryoprotective medium on the sperm, with dilution occurring over approximately 20 s, which was completed zero to two minutes post-thawing (mean (±SEM) 1.57 ± 0.057 min post-thaw). Activation treatments occurred simultaneously, with two researchers diluting aliquots of the thawed sample at the same time. Samples were then incubated for a minimum of five minutes to allow for motility activation before assessment using the CASA system. Samples were loaded onto a disposable semen analysis slide (SC20-01-04-B, 20 µm depth, Leja, Niew-Vennep, The Netherlands) one minute before assessment.

Activated samples were then assessed one at a time, with the order of activation treatments alternated between straws (i.e., control followed by caffeine treatments for straw 1, then caffeine followed by control treatments for straw 2, etc.). The first activation treatment was assessed five minutes post-activation, and the second activation treatment was assessed six to 13 min post-activation (mean (±SEM) 7.82 ± 0.22 min post-activation). Post-thaw sperm viability and motility characteristics were assessed simultaneously.

### 2.7. Statistical Analysis

To evaluate the effect of BSA addition to the cryopreservation medium, and caffeine addition to the activation medium, on post-thaw sperm characteristics, linear mixed-effects models (LME) were used. Before analysis, assumptions of normality were tested for each response variable using Shapiro–Wilk tests. Because data were not normally distributed, post-thaw sperm viability, post-thaw sperm total motility, and post-thaw sperm FPM were transformed using an arcsine square root transformation (sin^−1^[√x]), and post-thaw sperm VAP and post-thaw sperm VCL were transformed using a natural log transformation.

To determine the effect of BSA treatment (0%, 0.5%, 1% BSA) on sperm viability, an LME model was performed, with treatment as a fixed factor, and the response variable was post-thaw sperm viability, with frog ID included as a random effect and pre-freeze sperm concentration included as a co-variate.

To determine the effect of BSA-activation treatment (CPA (control) 0% BSA, control activation; CPA (control) 0% BSA, caffeine activation; CPA + 0.5% BSA, control activation; CPA + 0.5% BSA, caffeine activation; CPA + 1% BSA, control activation; CPA + 1% BSA, caffeine activation) on sperm motility characteristics, four separate LME models were performed. Within each model, treatment was a fixed factor; the response variable was either % total motility, % FPM, VAP, or VCL; frog ID was included as a random effect, and pre-freeze sperm concentration and assessment time were included as co-variates. In the LME models, sperm concentration did not have a significant effect on any of the sperm characteristics, while assessment time was significantly correlated with all sperm motility characteristics. To further explore the relationship among the motility variables (%total motility, %FPM, VAP, and VCL) and assessment time (minutes post motility-activation), regression analyses were performed.

All statistical analyses were performed using JMP Pro 17.0 software (SAS Institute Inc., Cary, NC, USA). For all tests, statistical significance was accepted at *p* < 0.05.

### 2.8. Ethics Statement

All experimental procedures were approved by the Animal Ethics Committee of the Taronga Conservation Society Australia (approval number 4b0820).

## 3. Results

### Effects of BSA and Caffeine on Post-Thaw Sperm Characteristics

Mean percent sperm viability remained fairly high post-thaw, ranging from 64.6 to 65.2% across all three treatment groups. Across all treatment groups, mean (±SEM) relative viability was 69.9% (±0.77) (relative to pre-freeze measures). Overall, BSA supplementation during cryopreservation (0%, 0.5%, 1% BSA) did not significantly influence percent sperm viability (LME, *p* > 0.05; Table 3).

Overall, experimental treatment (CPA (control) 0% BSA, control activation; CPA (control) 0% BSA, caffeine activation; CPA + 0.5% BSA, control activation; CPA + 0.5% BSA, caffeine activation; CPA + 1% BSA, control activation; CPA + 1% BSA, caffeine activation) did not have a significant effect on any of the sperm motility characteristics (% total motility, % FPM, VAP, or VCL; LME, *p* > 0.05; Table 3). In each LME model, the assessment time covariate was significantly correlated with each of the motility characteristics. Subsequent regression analyses revealed that each motility measure was significantly correlated with assessment time (Table 4), with each sperm motility measure declining with increasing assessment time, where assessment time ranged from 5 to 13 min post-activation. For details, see Figure S1 in the Supplementary Materials. Across all treatment groups, mean (±SEM) relative total motility was 47.6% (±1.13), and mean (±SEM) relative FPM was 20.8% (±1.05) (relative to pre-freeze measures).

## 4. Discussion

The development of optimal protocols for the cryopreservation of amphibian sperm is essential to improving the quality of sperm post-thaw and subsequent outcomes of assisted fertilisation (AF). Despite the potential for BSA and caffeine to improve post-thaw sperm characteristics, this study is the first to empirically test supplementation with these compounds in a threatened frog species. The results from this study found that neither the addition of BSA (0, 0.5, or 1%) to the cryopreservation medium nor the addition of caffeine (0 mM, or 4.5 mM) to the activation medium significantly affected any of the post-thaw sperm characteristics measured. These results therefore provide no evidence that supplementation with either BSA or caffeine improve sperm cryopreservation protocols for the Booroolong frog at the concentrations tested.

Despite reported benefits of BSA in other taxa [34,38,39,40,41], supplementation of cryopreservation media with BSA did not produce a significant effect on sperm motility or viability in the present study. The ability for BSA to minimise cryoinjury is theorised to be two-fold; firstly, it may act as an antioxidant [36,74], and secondly, it may increase membrane fluidity [75]. Compared to somatic cells, sperm cell membranes have higher levels of polyunsaturated fatty acids (PUFAs) and lower levels of cholesterol. Whilst these PUFAs are essential for membrane fluidity [76], they contribute to the sperm membrane being more susceptible to lipid peroxidation and oxidative stress which occurs during the cryopreservation process [77,78,79,80]. In addition to this, sperm cells typically produce high levels of ROS and have low antioxidant capacity [81], with these effects increasing during the freeze–thaw process [24]. Therefore, the addition of antioxidants to cryopreservation media to scavenge ROS and minimise lipid peroxidation has been investigated as a way of minimising this damage in a range of taxa [24,25,27,31]. An increase in sperm motility and viability following supplementation with BSA has been reported in a number of species and has been attributed to its antioxidant action, primarily by increasing the activity of endogenous antioxidants [33,36]. The absence of significant effects in the current study may be due to species-specific differences in both ROS accumulation and naturally occurring antioxidants in the sperm and seminal plasma. Currently, the level of oxidative stress experienced by amphibian sperm during storage is unknown, as are the endogenous antioxidants present in sperm and seminal plasma [16], so future studies quantifying these variables would be informative.

Another reason why BSA may not have improved post-thaw sperm metrics in the Booroolong frog relates to sperm membrane composition. High membrane fluidity may contribute to cryoinjury resistance, as this is essential for the maintenance of sperm function [80], and whilst PUFAs increase membrane fluidity, higher levels of cholesterol increase membrane rigidity [82]. In species with high levels of cholesterol, supplementation with BSA, which binds to the sperm membrane [83] and removes cholesterol from it, can therefore increase membrane fluidity and subsequently improve post-thaw outcomes [84]. As such, interspecific differences in the sperm membrane composition may explain a lack of significant differences found following BSA supplementation in the present study. Whilst there is currently little available knowledge on the sperm membrane composition for amphibians, research with mammals and fish has demonstrated high inter-specific diversity in sperm membrane compositions. For example, in mammalian sperm membranes, the content of docosahexaenoic acid (DHA), one of the key PUFAs, has been shown to vary greatly among species [85], while in fish, phospholipid composition has been found to exhibit interspecific variation [86]. Sperm membrane composition may also be differentially affected by the cryopreservation process, which can alter the lipid profile [87], and in some species, this alteration can be mitigated by additives that protect the membrane such as BSA [41]. Differences in sperm membranes may be, in part, due to differences in the fertilisation environment among species. In a study of the sperm membrane lipid composition of two freshwater fish species, the internally fertilising river stingray (*Potamotrygon motoro*) had a higher cholesterol content than the externally fertilising sterlet (*Acipenser ruthernus*), and phospholipid composition also showed marked differences across these species [88]. In an amphibian, the yellow-spotted mountain newt, addition of 5% BSA to the cryopreservation medium was associated with higher motility compared to the addition of 1% BSA; however, this was only true when the concentration of DMSO was 10% (comparatively, motility was 0% when DMSO was 5%) [46]. This may, in part, be due to this species’ reproductive mode of internal fertilisation. It may be that externally fertilising freshwater species have a naturally heightened sperm membrane fluidity to tolerate the swelling induced by exposure to the hypoosmotic environment, in which case the efflux of cholesterol by BSA may offer no additional benefit. Whether this pattern applies to amphibians remains to be determined and future studies investigating the membrane composition and how this differs among species are needed. Additionally, a beneficial effect of the addition of BSA was only observed when the concentration was 5%, which is comparatively much higher than what was tested in the present study (0.5 and 1%). Finally, as BSA is a mammalian-derived compound, there may be benefit in testing supplementation with taxa-specific proteins that may interact differently with the sperm membrane.

Contrary to expectations, the addition of the PDE inhibitor caffeine to the activation medium also had no effect on post-thaw sperm motility. Several studies spanning a diversity of taxa, including mammals [54,55], reptiles [59], fish [63], and corals [60,61], have reported beneficial effects of caffeine on post-thaw sperm motility. While these studies clearly demonstrate the potential for caffeine to improve motility activation, several other studies have reported no effect of caffeine supplementation [39,62,89,90]. This discrepancy may be due to species-specific differences in optimal caffeine concentrations, or species-specificity in the resilience of sperm to ROS. The concentration of caffeine used in the present study (4.5 mM) may have been sub-optimal for our study species. In several previous studies, it was found that sperm motility increased in a dose-dependent manner as PDE inhibitor concentration increased, including research in the cane toad [64], several species of fish [62,63], and in human and non-human mammals [91,92]. While no amphibian studies have previously activated frozen–thawed sperm with PDE inhibitors, a small number of studies have applied PDE inhibitors to fresh amphibian sperm, with varying results. In the cane toad (*Rhinella marina*), activation in optimal osmolality with 5 mM theophylline resulted in the highest motility compared to the control treatment [64]. In contrast, the addition of the PDE inhibitors caffeine, theophylline, and pentoxifylline at a concentration of 3 mM to fresh testicular macerates of the Puerto Rican coqui (*Eleutherodactylus coqui*) did not improve sperm motility, and caffeine at this concentration had a negative effect on sperm motility [65]. A previous study in the Booroolong frog found no effect of 2.5 mM or 5 mM of caffeine, theophylline, or pentoxifylline on initial sperm motility (though delayed effects were observed several hours post-activation, see below). Of note, these studies assessed the effect of PDE inhibitors on fresh sperm, and as such, the optimal doses may differ compared with post-thaw samples. Additionally, optimal doses would likely be partially dependent on the resulting osmolality of the activating solution, as higher caffeine concentrations may in turn increase the osmolality and lower motility activation [62]. As such, the relationship between the optimal dose of PDE inhibitors and sperm motility may not be linear, and is also likely to be species-specific. Therefore, there may be reason to test post-thaw motility activation in a broader range of caffeine concentrations, or by testing the effect of other PDE inhibitors, such as theophylline or pentoxifylline.

The lack of significant effect of caffeine supplementation to the activation media in the present study may also be due to Booroolong frog sperm being naturally resilient to ROS. Whilst ROS production may increase during the freeze–thaw process [24], frozen sperm cells should be rendered metabolically inactive, and thus ROS is not expected to accumulate during frozen storage, and resumption of ROS production should only occur upon thawing. At the time of motility assessment, there may have been limited ROS and so the antioxidant action of caffeine may have been negligible. In a previous study in the Booroolong frog conducted on fresh (unfrozen) sperm, supplementation of activation media with the either 2.5 mM caffeine or theophylline showed a delayed improvement to sperm motility, possibly due to the antioxidant action following the accumulation of ROS over time [66]. Sperm of the Booroolong frog exhibit extreme sperm longevity post-motility activation compared with other frog species [66], and it has been suggested that endogenous antioxidant action may be sufficient to limit oxidative stress until several hours after activation in this species [93]. Caffeine supplementation to activation media may have more beneficial effects on sperm performance in species with shorter sperm longevity post-activation and/or greater ROS sensitivity. To explore this possibility, it would be valuable to investigate ROS accumulation in post-thaw spermic urine samples and associated changess with antioxidant supplementation, in particular in species that exhibit limited sperm longevity.

Whilst the present study revealed no significant differences following supplementation with either BSA or caffeine, it is possible that effects may not have been detected due to variation in post-thaw assessment time. The present study found a significant negative correlation between assessment time and sperm motility characteristics, regardless of treatment. It is known that amphibian sperm motility decreases with increasing time post-activation [94]; however, it was unexpected that a significant decrease in motility characteristics would occur over such a short time-period, especially given the extreme longevity of sperm motility reported for fresh sperm samples of our study species [66], as well as the absence of a significant decline in post-thaw motility in a previous study [50]. The speed at which motility declines post-activation may be more pronounced in frozen–thawed sperm, compared to fresh sperm, due to damage induced by cryoinjury and exposure to cytotoxic CPAs, with different CPAs also likely influencing the speed of decline differently. Due to the significant correlation between assessment time and sperm motility metrics in the present study, it is possible that subtle treatment effects may have remained undetected due to variation in assessment time and elevated variance in sperm performance within treatments. A similar study quantifying the effects of various CPAs on post-thaw sperm quality in the yellow-spotted treefrog *Litoria castanea* also exhibited significant correlations between assessment time and sperm motility characteristics (Silla et al., unpublished data). Taken together, the results of the present study with those of the yellow-spotted treefrog study highlight that future studies should ensure that sperm motilities are assessed shortly following activation, and that post-activation assessment time is kept consistent across experimental treatments. The decline of sperm motility characteristics of frozen–thawed sperm post-activation reported in the present study may have additional implications for assisted fertilisation procedures, whereby fertilisation success may also decline with increased time post-thaw, and this also warrants further investigation.

## 5. Conclusions

The present study investigated the effect of BSA supplementation pre-freeze, and caffeine supplementation post-thaw, on sperm characteristics following cryopreservation in the Booroolong frog. There was no evidence of beneficial effects of either additive on any of the sperm characteristics tested. Future studies would benefit from exploring sperm membrane composition, ROS production during storage, naturally occurring antioxidants, and motility-activating pathways in amphibian sperm, where knowledge is currently lacking. Gaining knowledge in these areas will help to inform and direct protocol optimisation in our study species and other threatened amphibian species globally. Finally, we also recommend that future studies ensure that sperm performance is assessed rapidly post-thawing to avoid temporal effects on sperm characteristics.

## Figures and Tables

**Table 1:** Sperm quality characteristics of spermic urine samples (*n* = 14 for sperm concentration and viability; *n* = 12 for motility, forward progressive motility (FPM), sperm average path velocity (VAP), and sperm curvilinear velocity (VCL)) pre-freeze.

Sperm Characteristics (Pre-Freeze)	Mean	±SEM
Sperm concentration (×10^6^ sperm cells mL^−1^)	13.98	±2.22
Viability (%)	93.14	±1.53
Total motility (%)	94.07	±1.29
FPM (%)	89.28	±2.45
VAP (µms^−1^)	25.66	±2.46
VCL (µms^−1^)	55.09	±4.69

**Table 2:** The composition, pH, and osmolality of the three cryopreservation media containing different levels of bovine serum albumin (BSA).

	0% BSA (Control)	0.5% BSA	1% BSA
DMF (*w*/*v*)	20%	20%	20%
Trehalose (*w*/*v*)	20%	20%	20%
BSA (*w*/*v*)	-	0.5%	1%
pH	7.34	7.39	7.35
Osmolality (mOsmol kg^−1^)	990.5	1001.0	1217.5

NOTE: Each cryoprotectant solution contained equal concentrations of dimethylformamide (DMF) and trehalose (TRE) diluted in SAR. The concentrations provided are those in the initial solution, before 1:1 (*v*/*v*) dilution with the sperm sample; final concentrations in solution within each cryostraw were therefore half of those concentrations presented above. The pH values of the cryopreservation media were measured using a pH meter (Orion Star A211 pH meter, Thermo Fisher Scientific, Waltham, MA, USA), and osmolality was measured using a vapour pressure osmometer (Vapro^®^ model 5600 Wescor Inc., South Logan, UT, USA); osmolality measures were duplicated and averaged.

**Table 3:** Effect of BSA activation treatment (CPA (control) 0% BSA, control activation; CPA (control) 0% BSA, caffeine activation; CPA + 0.5% BSA, control activation; CPA + 0.5% BSA, caffeine activation; CPA + 1% BSA, control activation; CPA + 1% BSA, caffeine activation) on post-thaw sperm characteristics (viability; total sperm motility; forward progressive sperm motility, FPM; sperm average path velocity, VAP; and sperm curvilinear velocity, VCL).

Sperm Characteristics (Post-Thaw)	Treatment						*F_df_*	*p*
	CPA + 0% BSA	CPA + 0% BSA	CPA + 0.5% BSA	CPA + 0.5% BSA	CPA + 1% BSA	CPA + 1% BSA		
	Control Activation	Caffeine Activation	Control Activation	Caffeine Activation	Control Activation	Caffeine Activation		
Viability (%)	65.00 ± 2.14	-	64.61 ± 1.78	-	65.21 ± 1.29	-	0.0386 _2,26_	0.9622
Total motility (%)	42.59 ± 2.74	44.81 ± 2.81	44.59 ± 2.43	45.85 ± 2.58	43.76 ± 3.70	41.91 ± 2.59	0.4710 _5,64.09_	0.7965
FPM (%)	17.72 ± 2.13	20.04 ± 2.76	19.83 ± 2.52	20.62 ± 2.71	17.49 ± 2.76	17.50 ± 2.39	0.9894 _5,64.03_	0.4313
VAP (µm s^−1^)	2.51 ± 0.30	2.54 ± 0.27	2.49 ± 0.20	2.65 ± 0.27	2.42 ± 0.26	2.21 ± 0.20	0.9593 _5,64.04_	0.4495
VCL (µm s^−1^)	5.93 ± 0.59	6.19 ± 0.59	6.11 ± 0.44	6.40 ± 0.54	5.68 ± 0.53	5.38 ± 0.45	1.3050 _5,64.04_	0.2730

Data shown are untransformed mean ± SEM (*n* = 14 per treatment). *F_df_* and *p* values displayed are the results of LME models on transformed data.

**Table 4:** Effect of time post-activation (mins) on post-thaw sperm characteristics (total sperm motility; forward progressive sperm motility, FPM; sperm average path velocity, VAP; and sperm curvilinear velocity, VCL).

Sperm Characteristics (Post-Thaw)	r^2^	*F_df_*	*p*
Total motility (%)	0.10	9.47 _1,82_	0.0028
FPM (%)	0.11	10.26 _1,82_	0.0019
VAP (µm s^−1^)	0.12	10.99 _1,82_	0.0014
VCL (µm s^−1^)	0.13	11.80 _1,82_	0.0009

*F_df_* and *p* values displayed are the results of LME models on transformed data.

## Data Availability

The data presented in this study are available on request from the corresponding author. The data are not publicly available, in accordance with Taronga Conservation Society Australia’s policies on data and sample sharing (Opportunistic Sample Request Policy).

## Supplementary Materials

The following supporting information can be downloaded at https://www.mdpi.com/article/10.3390/vetsci12010030/s1; Figure S1: Effect of time post-activation on post-thaw sperm (A) total motility, (B) forward progressive motility, (C) average path velocity (VAP) and (D) curvilinear velocity (VCL) in the Booroolong frog *Litoria booroolongensis*. Data points shown are each activated sample that were assessed, across both activation treatments (MilliQ water alone and MilliQ water with 4.5 mM caffeine). R^2^ values shown are the result of regression analyses.
