# Supplementary material for: Effect of Bovine Serum Albumin (BSA) Concentration on Cryopreservation of Booroolong Frog Sperm with Evaluation of Post-Thaw Motility in Caffeine
Source: Vet Sci. 2025 Jan 8;12(1):30. doi: 10.3390/vetsci12010030 (PMC11769386; doi:10.3390/vetsci12010030)
Supplement: Supplementary file 1 [file vetsci-12-00030-s001.zip › vetsci-3355728-supplementary.pdf]

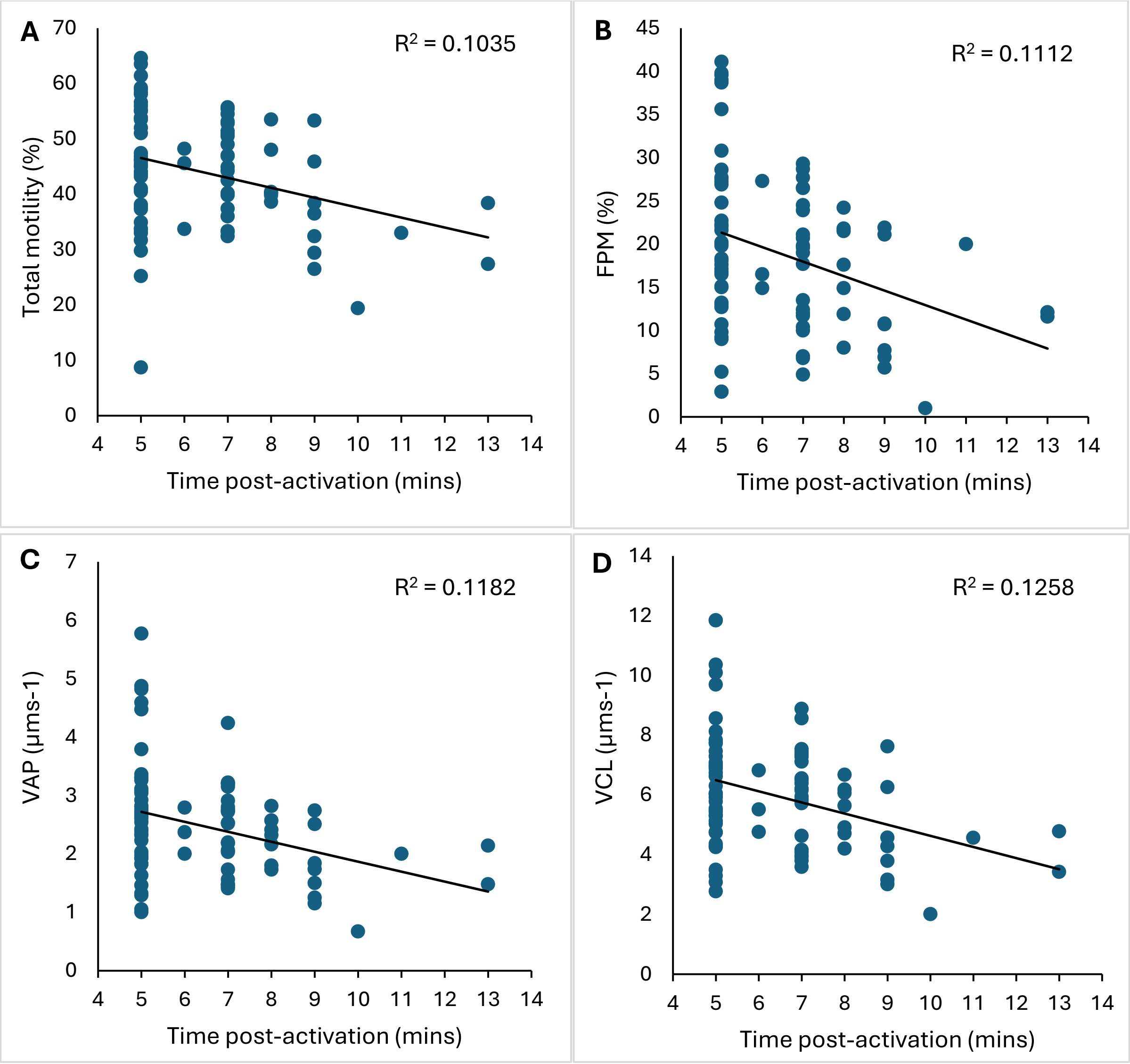

Supplementary Figure S1: Effect of time post-activation on post-thaw sperm (A) total motility, (B) forward progressive motility, (C) average path velocity (VAP) and (D) curvilinear velocity (VCL) in the Booroolong frog *Litoria booroolongensis*. Data points shown are each activated sample that were assessed, across both activation treatments (MilliQ water alone and MilliQ water with 4.5mM caffeine). R^2 values shown are the result of regression analyses.
